# Malignant Skin Cancer Excision in Combined Therapy with Electro-Chemotherapy and Dermal Substitute

**DOI:** 10.3390/curroncol28030160

**Published:** 2021-05-05

**Authors:** Barbara De Angelis, Alberto Balzani, Alessia Pagnotta, Eleonora Tati, Fabrizio Orlandi, Margarida Fernandes Lopes Morais D’Autilio, Valerio Cervelli, Pietro Gentile

**Affiliations:** 1 Surgical Science Department, Medical School, University “Tor Vergata”, 00133 Rome, Italy; bdeangelisdoc@gmail.com (B.D.A.); valeriocervelli@virgilio.it (V.C.); 2 Plastic and Reconstructive Surgery, Complex Operative Unit, “Policlinico Casilino”, 00169 Rome, Italy; balzanialberto@tiscali.it (A.B.); ele.tati@gmail.com (E.T.); fabrizio.orlandi86@gmail.com (F.O.); mflmdautilio@gmail.com (M.F.L.M.D.); 3 Hand and Microsurgery Unit, Jewish Hospital, 00148 Rome, Italy; pagal@interfree.it

**Keywords:** squamous cell carcinoma, malignant skin tumor treatment, electrochemotherapy, dermal substitute, skin cancer treatment

## Abstract

Squamous cell carcinoma (SCC) is the second most common malignancy skin cancer. It is characterized by abnormal, accelerated growth of squamous cells (SCs). SCC occurs when DNA damage from exposure to ultraviolet radiation or other damaging agents trigger abnormal changes in the SCs, presenting as painless lesions on areas of high sun exposure, such as the dorsum of the hand and upper extremity. For most skin SCC, the surgical excision alone is standard practice. However, recent efforts in new treatment strategies have involved around adjuvant or concomitant electrochemotherapy (ECT). ECT is a non-thermal tumor ablation modality, safe and effective on any type of solid tumor. An 87-year-old patient affected by hand SCC with invasion of deep structures including tendons was treated with neoadjuvant intra-tumoral ECT sessions followed by a selective surgical removal and reconstruction of the substance loss with collagen dermal template (CDT). Two neoadjuvant intra-tumoral ECT procedures, at distance of 3 months, with the aim to reduce the tumor size before a selective surgery, were performed. This study shows that combined surgical selective excision with ECT and CDT is a valid technique for the extended-deep dorsal hand tumor lesions reconstruction.

## 1. Introduction

Skin cancers represent the most common primary malignancies of the hand. Squamous cell carcinoma (SCC) is the second most common malignancy skin cancer [1]. SCC is characterized by abnormal and accelerated growth of squamous cells (SCs). They typically present as painless lesions on areas of high sun exposure, such as the dorsum of the hand and upper extremity [2]. SCC contributes substantially to morbidity and mortality among elderly persons [3,4,5,6] and it is caused by cells’ DNA mutation. It is one of the most common malignancy skin cancers and its incidence increases more rapidly with age [5,6,7]. The most important etiological factors include ultraviolet (UV), ionizing radiation, and certain chemical carcinogens. The risk factors that lead to SCC development can be divided into extrinsic (UV-A, ionizing radiation, human papilloma virus (HPV), and chemical substances) and intrinsic (genodermatoses, immunosuppression, preexisting skin lesion, and preexisting actinic keratosis) [6,7]. Often, SCC develops on areas of the body exposed to UV radiation either from sunlight or from tanning beds or lamps, although it can develop anywhere [8,9]. The cancer may spread to other areas of the body, including the lymph nodes and organs [10,11,12,13]. Once this occurs, the condition can be life-threatening. For most skin SCC, the surgical excision alone is considered the gold standard practice [13,14,15,16,17]. However, recent efforts in new treatment strategies have involved around adjuvant or concomitant electrochemotherapy (ECT). ECT is a non-thermal tumor ablation modality, safe and effective on any type of solid tumor [14,15,16,17,18,19]. We present the case of an 87-year-old patient affected by hand SCC with invasion of deep structures including tendons. The patient refused the possibility of limb amputation as a first line therapy, proposed by our institute. Therefore, the surgical team decided to treat the lesion with neoadjuvant intra-tumoral ECT with bleomycin sessions [20,21,22,23,24,25], as a preservative treatment, in order to obtain a reduction in the tumor size and then to proceed with the selective surgical removal and substance loss reconstruction with collagen dermal substitute template (CDT). A specific written informed consent was provided before the treatment. This study confirms the efficacy and safety of combined treatment and its benefits. Therefore, selective surgical tumor excision combined with ECT and CDT is a valid technique for extended-deep dorsal hand tumor lesion reconstruction. Based on our clinical case report, we can affirm that the combined technique is ideal for elderly patients, it reduces National Sanitary System (SSN) costs, and it is cost-effective compared to radical amputation, therefore increasing the patient’s quality of life.

## 2. Materials and Methods

The study protocol, object of an inter-university master’s degree called “*Regenerative surgery of loss of substance*”, was approved according to the Rectoral Degree (D.R. n. 1693/2016) of 12 July 2016, and the Ethics on Research Committee of the School of Medicine, “La Sapienza” University, Rome, Italy, and School of Medicine, “Tor Vergata” University, Rome, Italy, with registration number #27696, which is where clinical activities were performed. The patient received detailed oral and written information about the study, including the risks, benefits and alternative therapies, and signed an informed consent form before any study procedures.

### 2.1. Case Report

The patient was an 87-year-old female, affected by hand squamous cell carcinoma with invasion of deep structures including tendons. The patient had a positive history for recurrent sun burns on the hand dorsal zone. Patient showed at the Plastic and Reconstructive Surgery Department at “Tor Vergata” University, two years after acute enhancement of papular lesions on the dorsal non-dominant hand. At the time of our clinical evaluation, the lesion was superficially ulcerated, appeared to be approximately 5 × 4 cm in size, and had infiltrated the deep tendons. Although limb amputation was recommended, the patient refused this surgical treatment. On this basis, a multidisciplinary team composed of plastic surgeons, a dermatologist and a hand surgeon evaluated the lesion’s extension and depth, and suggested two neoadjuvant intra-tumoral ECT procedures, at a distance of 3 months, with the aim of reducing the tumor size before a selective surgery, as alternative to the limb amputation. The substance loss reconstruction was performed in a one-step surgery with selective surgery using a CDT. Biopsy confirmed the clinical diagnosis of SCC. Computed tomography (CT) and magnetic resonance imaging (MRI) were performed before the treatment, showing tumor invasion of deep and tendinous structures, and again after the procedures. The safety margins for complete eradication of the carcinoma were therefore represented by a partial amputation of the first, second and third distal and proximal phalanxes resulting in loss of the hand’s prehension capability. 

### 2.2. Combined Selective Tumor Excision with Electrochemotherapy and Collagen Dermal Substitute Template

**Figure 1:**
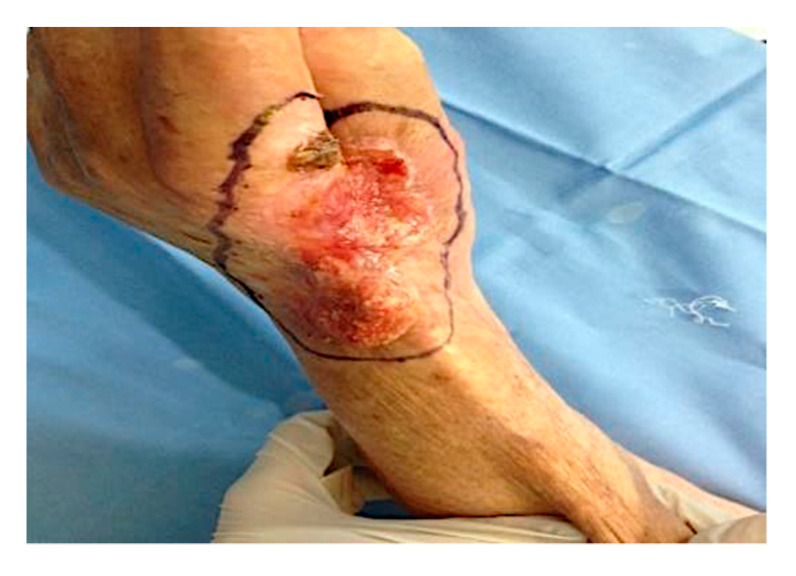
The surgical excision area, with healthy margins of 1 cm, included the area of the left radial carpus, and the first and second finger (total area 9.5 × 5 cm).

**Figure 2:**
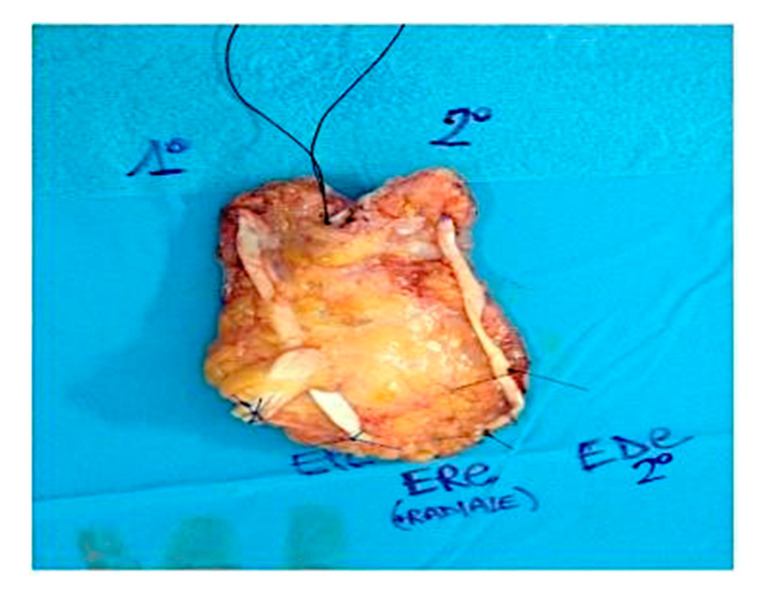
Intra-operative situation showing the excised Squamous Cell Carcinoma including a portion of the EPL, EDC and ECRL tendons.

**Figure 3:**
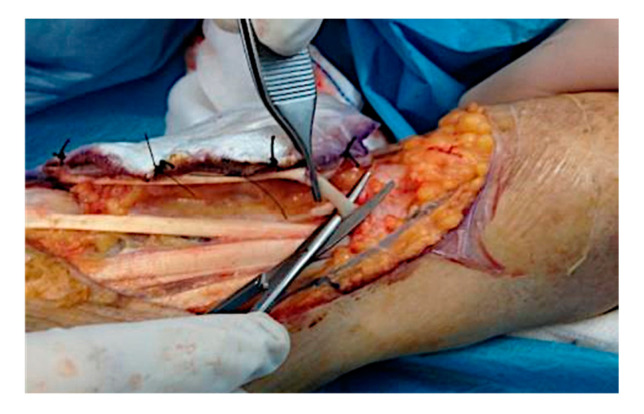
Intra-operative situation: isolation of the tumor area and excision of the lesion in the radioulnar direction with the sensory radial nerve of the tendon EPL, tendon EDC of the second finger and of the ECRL, remaining adherent to the skeletal plane.

The patient underwent two ECT sessions before selective surgery. The first ECT session was performed by administering an intravenous infusion of 24.5 mg of bleomycin in 60 s and by positioning hexagonal electrodes and releasing 40 discharges from the periphery to the lesion center. After 3 months, a second ECT session was performed. After infusion of 24.5 mg of bleomycin in 60 s, 25 discharges were released from the periphery to the lesion center. The surgical removal area of the lesion, with healthy margins of 1 cm, covered the radiocarpal wrist and the first and second finger area (total area 9.5 × 5 cm) (Figure 1). Prior isolation of the tumor area was performed. Lesion excision was performed, following the radioulnar track of the radial nerve sensitive branch, with surgical selective tumor removal up to the extensor pollicis longus (EPL) tendon on the first finger, the extensor digitorum communis (EDC) tendon at the second finger and the extensor carpi radialis longus (ECRL) tendon, remaining adherent to the skeletal plane (Figure 2 and Figure 3). 

**Figure 4:**
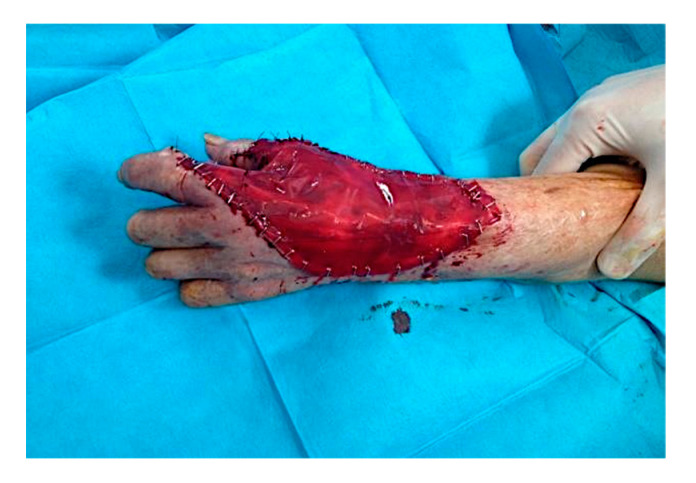
Intra-operative situation: a large sheet of CDT was placed to cover the loss of substance (area 12.5 × 7 cm).

A large sheet of CDT was placed in order to cover the substance loss. The area was 12.5 × 7 cm (Figure 4). After the engraftment of dermal substitute, at around 4 weeks, the CDT cover silicone layer was removed, and the area was grafted with a thin epidermal skin graft. Therefore, selective surgical excision of the lesion was carried out by saving the rays and with minimal damage to the apposition movements, and then covered with regenerative dermis and dermal epidermal graft.

### 2.3. Follow-Up Protocol

Clinical, histological and instrumental evaluations were performed over a follow-up period of three years. Clinical evaluations were performed every 3 months. Ultrasonography of nodal stations was performed at the time of diagnosis and 6 months after the treatment. MRI was performed after 6 months and then every year. A punch 4 mm biopsy was performed 1 year after the treatment, confirming the absence of a relapse.

## 3. Results

After the second ECT session, a new MRI was compared to that taken before the treatment, resulting in a resolution of tendinous involvement from the tumor mass and a partial reduction in the lesion size.

**Figure 5:**
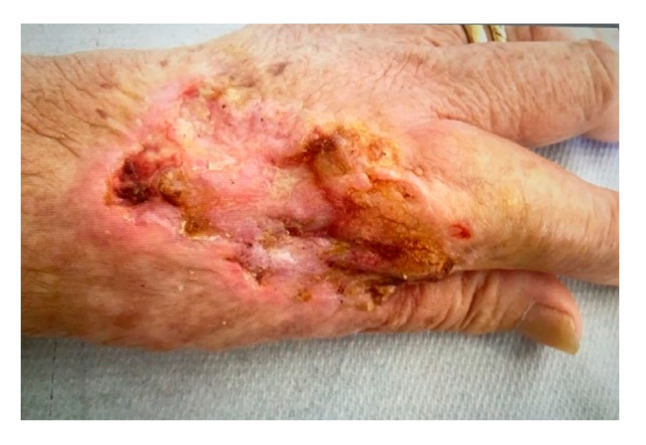
The picture shows the tissues situation 4 weeks after the second ECT treatment. Note the entity of the margin reduction of the primary lesion with evident fibrosis of the re-epithelialized portion. A lesion size reduction of 40% was observed.

In detail, after two ECT sessions with bleomycin were performed, a lesion size reduction of 40% (30% at the first session and 10% at the second) was observed (Figure 5).

**Figure 6:**
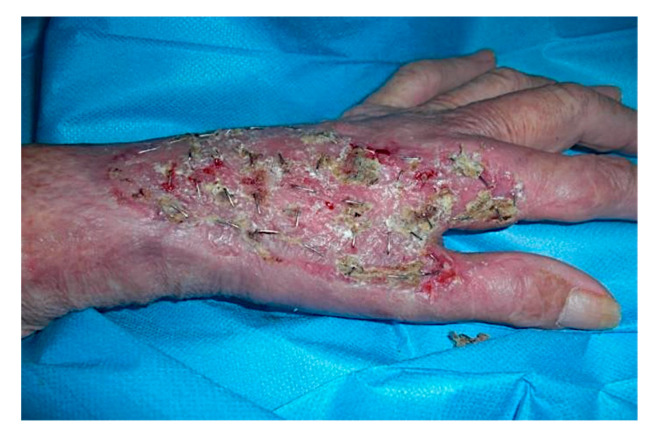
Post-operative situation after 10 days. The left hand dorsum treated with CDT and successive skin grafting showing re-epithelialization.

These results allowed us to remove the tumor lesion without causing the demolition of any fingers, and without causing a deficit in the hand prehension capability, but only in the fingers’ abduction/adduction for the detriment of the sensitive radial nerve, the thumb extensor tendon, the second finger common extensor tendon and the extensor carp radialis longus. The ECT avoided the limb amputation, which was refused by the patient, in any case. Four weeks after the CDT engrafting, the covering silicone lamina was removed, and the area was grafted with a thin dermo-epidermal graft. The healing time was around 10 days (Figure 6).

The ECT combined with selective surgical excision, CDT and skin graft meant avoiding the mobilization of a large-microsurgical flap in a cardiopathic and smoking elderly patient. Four different stages of dermal regeneration could be demonstrated histologically: imbibition, fibroblast migration, neovascularization and remodeling and maturation. Full vascularization of the neo-dermis occurred at four weeks. An amplified range of movement and improvement in appearance compared with her preoperative states were observed. It seems that matrix color reflected the step of neo-dermal vascularization. Elastic fibers were seen in our specimen even though they were of abnormal morphology. Nerve fiber regeneration was found in this specimen, although it was confined to the mid or lower reticular dermis.

## 4. Discussion

SCC is a non-melanocytic skin cancer (NMSC) type. It is a common hand malignancy; however, recurrence rates, metastatic rates, and long-term survival rates have not been well defined [13]. NMSC is the most common malignancy found in humans. The incidence rate is thought to double in the next 30 years [16]. Incidence also increases with age. According to several studies, incidence of basal cell carcinoma (BCC) in individuals over 75 years old was approximately five times higher compared to individuals between 50 and 55 years old, and for SCC, approximately 35 times higher [18,26,27,28,29,30]. SCCs can occur on all areas of the body, including mucous membranes and genitals, but are most common in areas frequently exposed to the sun, such as the ear rim, lower lip, face, balding scalp, neck, arms, legs and hands. Often the skin in these areas reveals some signs of sun damage, including wrinkles, pigment changes, freckles, “age spots”, loss of elasticity, and broken blood vessels.

Post-oncologic dorsum-hand deficiency reconstruction, particularly in elderly patients with multiple comorbidities, presents a challenge for the plastic surgeon. Several clinical studies have shown that ECT can be used in any type of solid tumor, while CDT can be used to repair severe hand wounds and defects after tumor resections [8,14,19,29,30,31,32,33,34]. A speedy procedure with an acceptable aesthetic and functional outcome is required, especially in elderly patients. Histological analysis is relevantly important in documenting the healing process. Understanding the cellular process by which ECT associated to CDT, incorporated, and vascularized, can aid surgeons who frequently treat complex anatomical structures defects. CDT consists of a bilayer skin regeneration system [35]. The outer layer is a thin silicone film that acts as the patient’s epidermis. The inner layer is made of a complex matrix of cross-linked fibers that acts as a scaffold for the regeneration of dermal skin cells. CDT has been shown to be useful in treatment after the ECT session in large size tumor resection in the hand. After vascularization, the epidermal skin replacement is generated afterwards [29,31,35,36].

ECT with bleomycin increases intrinsic cytotoxicity, as it acts on the cellular DNA directly, giving good results in treatments to reduce tumor size. It has been stated in the literature that this procedure is also based on the achievement of in vivo tumor cellular electro permeabilization by means of electric pulses locally delivered to the tumors after bleomycin injection; cellular electro permeabilization can affect all tumor cell types, allowing the anticancer drugs—in this case, bleomycin—to enter the cells, thus greatly magnifying their cytotoxicity [15,16,19].

Regarding our clinical experience, the combined treatment based on ECT, with intravenous infusion of bleomycin, and CDT may be highly effective, therefore offering benefits such as safety, simplicity, a decrease in toxicity and, mainly, a reduction in the tumor mass, facilitating the successive selective surgery, and therefore avoiding major procedures, such as radical amputation (our patient had refused the possibility of amputation of the limb since the beginning, even having notable loss of hand’s prehension capability) [20,21,22,23,24,25].

ECT and CDT are effective combined techniques for dealing with large tumors and/or hand lesions [9,10]. This can potentially minimize the need for free-microsurgical flap coverage use and should be taken into consideration as a viable alternative in important hand reconstruction. The result obtained in this single case report was achieving durable, functional, and aesthetic permanent regeneration while, consenting a satisfying polydigital prehension.

As in all procedures, these techniques have advantages and disadvantages. The disadvantages are the necessity for two ECT sessions, and the risks of infection under the silicone layer, the silicone becoming detached or the recurrence of contraction. However, the infection can be easy controlled through antibiotic prophylaxis. On the other hand, combined techniques have many advantages, including the immediate accessibility, simplicity and consistency of the CDT, and the malleability and cosmetic appearance of the resulting cover. The ECT not only gives positive antitumor effects, but also gives better cosmetic results than excisional surgery [15].

The Mohs micrographic surgery was not indicated in the clinical case treated, because it is used when a radical removal of a tumor lesion is performed, helping to specify its extent and aiming to proceed correctly with complete excision. The treated lesion was very extensive in depth, with involvement of tissues without metastases, and according to the guidelines and the articles analyzed [20,21,22,23,24,25], it was a candidate for amputation. The patient refused this intervention, and, for this reason, a combined treatment was chosen after ECT.

Combined treatment with ECT and CDT is an alternative technique for extended dorsum hand tumor lesion reconstruction. It can be conducted without major complications, obtaining a short healing time with acceptable cosmetic results. It is a multidisciplinary approach, and it is ideal for elderly patients. Therefore, this study confirms the efficacy and safety of the combined treatment, and its benefits. Furthermore, based on our clinical case, we can confirm that combined techniques reduce National Sanitary System costs, and that it is cost-effective compared to radical amputation, therefore increasing the patient’s quality of life.

## 5. Conclusions

The SCC eradication with ECT is currently very much debated and it is considered the gold standard only for BCC treatment. The use of ECT neoadjuvant therapy, for a mass cytoreduction and its simple removal, proves to be reasonable also for SCC, in our opinion. Our 87-year-old patient refused the possibility of limb amputation from the beginning. She asked for a more conservative treatment. Her cardiac disorders and tobacco habit meant that a microsurgical flap reconstruction was not recommended, both due to the anesthesiologic risks and the necrosis possibility of a reconstruction flap. CDT permitted the covering of the substance loss after the selective tumor excision, confirming efficacy and minimal invasiveness. These techniques—ECT, CDT and selective tumor excision—combined may, in selected cases, lead to an eradication, reconstruction and maintenance of the limb functionality, which is difficult to obtain with the other techniques. The current three-year follow-up testifies the radical nature of what has been achieved.

## Data Availability

The data that support the findings of this study are available on request from the corresponding author, P.G. or from the first author B.D.A.
